# Predict the Relationship between Gene and Large Yellow Croaker’s Economic Traits

**DOI:** 10.3390/molecules22111978

**Published:** 2017-11-16

**Authors:** Xiangxiang Zeng, Shuting Jin, Jing Jiang, Kunhuang Han, Xiaoping Min, Xiangrong Liu

**Affiliations:** 1 School of Information Science and Technology, Xiamen University, Xiamen 361005, China; xzeng@xmu.edu.cn (X.Z.); stjin.xmu@gmail.com (S.J.); jingdefengye1314@126.com (J.J.); 2 State Key Laboratory of Large Yellow Croaker Breeding, Ningde Fufa Fisheries Company Limited, Ningde 352000, China; hankunhuang@foxmail.com

**Keywords:** large yellow croaker, KATZ, trait gene prediction

## Abstract

The importance of a gene’s impact on traits is well appreciated. Gene expression will affect the growth, immunity, reproduction and environmental resistance of some fish, and then affect the economic performance of fish-related business. Studying the connection between gene and character can help elucidate the growth of fishes. Thus far, a collected database containing large yellow croaker (*Larimichthys crocea*) genes does not exist. The gene having to do with the growth efficiency of fish will have a huge impact on research. For example, the protein encoded by the IFIH1 gene is associated with the function of viral infection in the immune system, which affects the survival rate of large yellow croakers. Thus, we collected data through the published literature and combined them with a biological genetic database related to the large yellow croaker. Based on the data, we can predict new gene–trait associations which have not yet been discovered. This work will contribute to research on the growth of large yellow croakers.

## 1. Introduction

The large yellow croaker (*Larimichthys crocea*), whose meat is delicious and has therapeutic effects, is a crucial source for the fish markets. Larger yellow croakers, belonging to Asian croakers, are economically important, however few studies focus on the genes related to economic traits of the large yellow croaker. With the development of molecular biology technology [1], such as genomics in recent years, research on important economic traits of aquatic animals has attracted much attention. Molecular theory on sex, growth, disease resistance, cold and hypoxia, and other traits of common aquatic organisms has made some achievements, and a large number of related functional genes have also been identified from the large yellow croaker. In 2016, Shen et al. [2] sequenced and characterized the melanoma differentiation-associated antigen 5 (LcMDA5), laboratory of genetics and physiology 2 (LcLGP2) and mitochondrial antiviral signaling protein (LcMAVS) from the large yellow croaker. In 2017, Zou et al. [3] characterized an IRAK4 orthologue from the large yellow croaker, named Lc-IRAK4. Liu et al. [4] identified and characterized the chemokine receptor genes CXCR2 (LycCXCR2), CXCR3 (LycCXCR3), and CXCR4 (LycCXCR4) of the large yellow croaker and so on. This study gives us more information on the gene expression in large yellow croakers. However, due to the limitations of the growth of research on large yellow croakers, previous studies only focused on individual genes’ expression and, until now, research on the gene–trait accession of this fish are still relatively scarce [5].

In order to systematically study the relationship between various traits and genes of the large yellow croaker, two contributions are made in this work. First, we collected large yellow croaker gene–trait associations that can affect economic activities related to the fish by searching the existing literature. Second, on the basis of these data, we predicted unknown gene–trait relationships. We collected papers related to the large yellow croaker through PubMed (https://www.ncbi.nlm.nih.gov/pubmed/) and the Baidu Encyclopedia (https://baike.baidu.com/). While collecting these papers, we filtered out the economic traits associated with the large yellow croaker. We used relevant genes to find data on the corresponding protein and protein sequence through Uniprot. The collected data contained the following information: reference, gene symbol/name, Uniprot Entry, protein sequence, trait, cause, pubmedID, chromosome location, and more.

KATZ [6] is a method based on network measurements which calculates the similarities between nodes in a heterogeneous network in order to solve the problem of association prediction. This method takes the number of walks and walk lengths between nodes in a graph into account as effective similarity measures. We translated the problem of gene–trait association measurement into the number of connections between trait and gene nodes in the heterogeneous network. With the collected experimental data, we constructed a large yellow croaker gene–trait association network and further used a new computational model of KATZ measurement for the large yellow croaker gene–trait association prediction (KATZ-YC), based on the assumption that functionally similar genes tend to have similar interaction and non-interaction patterns with trait, and vice versa [7]. To our best of knowledge, the KATZ-YC is the first tool created for the large yellow croaker gene–trait association prediction. The credible prediction performance could be owed to the use of KATZ measurements, and to the use of the Gaussian Interaction Profile (GIP) [8] kernel to obtain similarities for genes and traits. We propose a method that relies on the known gene–trait association network topology information, trait similarity network, and gene similarity network. The trait similarity network and gene similarity network were obtained by the GIP kernel [9]. LOOCV (Leave one out cross validation) and K-fold cross validation were performed in order to evaluate the validity of this novel computational model based on known gene–trait associations obtained from the data we collected. As a result, we obtained the LOOCV framework and 2-fold and 5-fold cross validation with average AUCs (Area under curve) of about of 0.7766, 0.7183 and 0.7476, respectively. It is anticipated that the KATZ-YC could be used to obtain more novel genes associated with important large yellow croaker traits, thereby facilitating the study of the genes and traits of the fish. Based on the existing research literature, we collected large yellow croaker gene–trait associations, including 24 traits and 102 genes. Importantly, the prediction accuracy was not ideal due to the lack of data. With the development of molecular technology, the relevant research will increase remarkably, making more date available, thus improving predictive performance. Moreover, this predicting method is more convenient and effective because it is completely based on known gene–trait associations.

## 2. Results

In this article, we collected the genes related to the economic traits of large yellow croakers. The existing studies on the genes and traits of large yellow croakers are still relatively few, and the related data is inadequate. We know that similar genes may contribute to similar traits. Based on this phenomenon and on the data we collected, new methods of computing status with KATZ [10] were developed in order to predict probable existing associations between genes and traits. We proposed the large yellow croaker gene–trait association prediction. In order to obtain similarity between genes and traits for the prediction of potential associations, we used the GIP kernel [11]. We used the KATZ-YC to predict associations between genes and traits. To obtain experimental results, we used LOOCV and K-fold cross validation. The KATZ-YC achieved a prediction performance with average AUCs of 0.7766, 0.7183 and 0.7476 when using the LOOCV framework and 2-fold and 5-fold cross validations, respectively. The prediction new genes that affect traits was achieved by using known gene–trait relationships that can help promote the survival rates of large yellow croakers and that can improve economic efficiency.

### 2.1. Leave-One-Out Cross Validation

We evaluated the performance of the KATZ-YC on gene–trait link prediction with the LOOCV method, based on the data we collected from various papers published about biological information. Many gene–trait associations from these data were correct. In the validation framework of LOOCV [12], each known gene–trait association was left out in turn for testing, and the other gene–trait associations were used as training samples for model learning. An important part of implementing this kind of validation is to obtain nearly all the samples for training, which results in a distribution of the training sample subjects similar to the distribution of the population samples. Also, there were no random factors affecting data during the experiment. Thus, the process of our validation is totally replicable. However, our research also had shortcomings. The cost of calculation was high because almost all the samples were used to train model for each round. When the sample population was large, LOOCV was difficult to implement in practice unless the training procedure for models was fast or was done in parallel in order to save time.

If the rank of a given testing sample was higher than the given threshold, the prediction was considered successful. We set different thresholds in order to obtain corresponding true positive rates (TPR, sensitivity) and false positive rates (FPR, 1-specificity) [13]. Thus, the receiver–operating characteristics (ROC) curve could be plotted by drawing TPR versus FPR at different thresholds. Areas under the ROC curve were calculated in order to evaluate the prediction performance of the KATZ-YC. A perfect prediction was an AUC value of 1. An AUC value of 0.5 indicated random performance [14]. 

**Figure 1:**
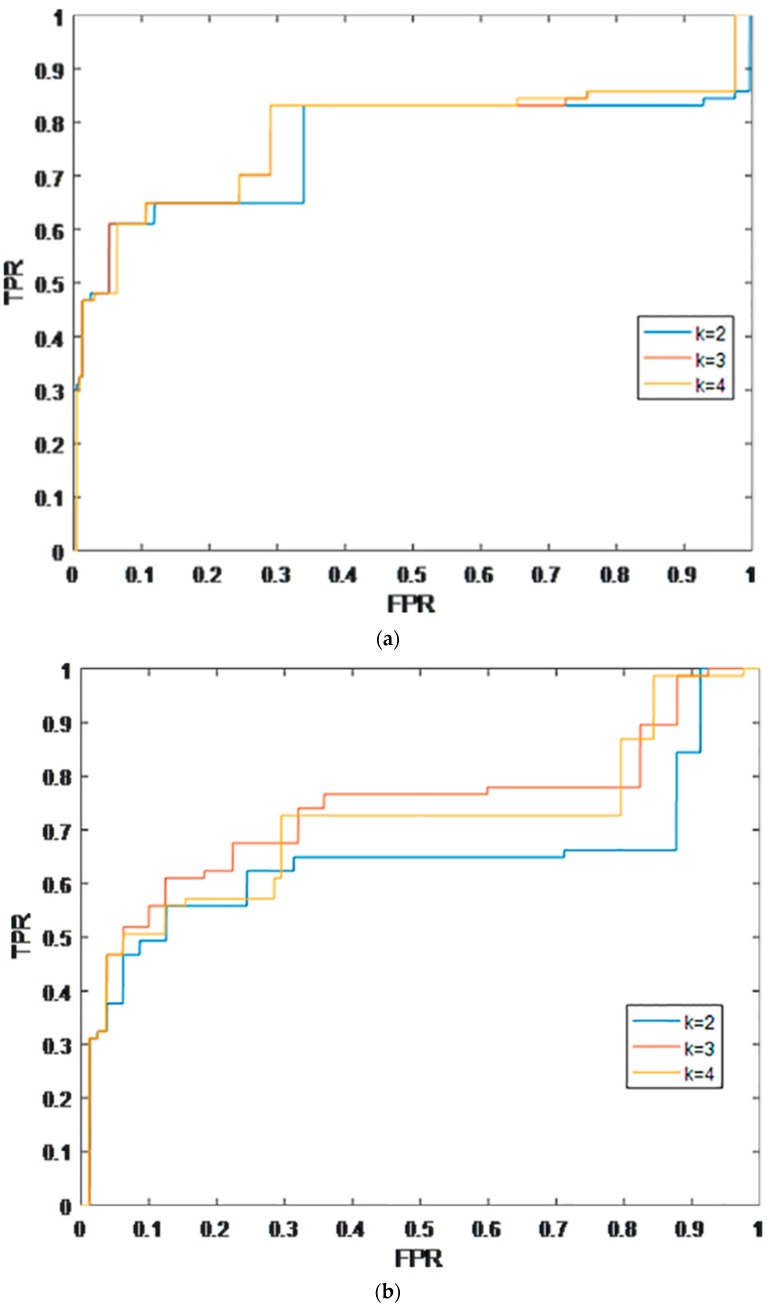

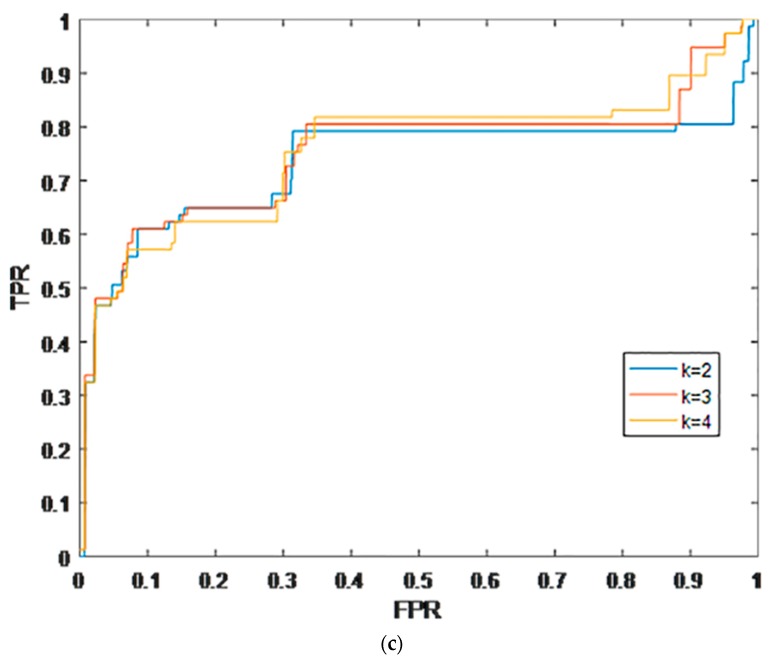
Prediction performance of KATZ-YC with different parameter settings (k = 2, 3 and 4) in terms of the receiver–operating characteristics (ROC) curve and area under the curve (AUC), based on K-fold cross validation: (**a**) based on Leave One Out Cross Valication (LOOCV); (**b**) based on 2-fold cross validation; and (**c**) based on 5-fold cross validation.

For the KATZ-YC, k is an important parameter, which represent the number of walks. The *k* value affected the prediction performance for gene–trait associations. We performed a series of comparative experiments in order to estimate the influence of k. As a result, the KATZ-YC achieved a better prediction performance when k was set as 2 (Table 1 and Figure 1a). Specifically, when k was set as 3, the KATZ-YC achieved AUC of 0.7766 in the framework of global LOOCV.

### 2.2. K-Fold Cross Validation

K-fold cross validation consists of dividing all samples into K copies. Generally, the number of each copy is equal or similar. One copy is taken as the test sample and the remaining K-1 as the training sample. This process repeats K times, and the final average test results can estimate the performance of the model. The final result was obtained by calculating the average result of these K results, or by using another way to obtain a single result. An advantage of implementing K-fold validation is that it randomly selects subsamples for training and validation repeatedly, and takes the results of each test into consideration. For further evaluation of the KATZ-YC prediction performance, we used 2-fold and 5-fold cross validation. Random sample division for performance evaluation can cause potential bias. As much as possible, in order to eliminate the potential bias, we divided the gene–trait associations many times and repeated experimental verification of the corresponding ROC curves. AUCs were obtained in a similar way as for LOOCV. The KATZ-YC achieved a prediction performance with average AUCs of 0.7183 and 0.7476 (with a standard deviation of 0.03) when using 2-fold (Table 2 and Figure 1b) and 5-fold cross validations (Table 3 and Figure 1c), respectively. We used the LOOCV and K-fold cross validation to test whether the method of the KATZ-YC prediction performance was trustworthy and valid. The gene–trait associations with higher ranks would be verified by experimental observations in the future.

## 3. Materials and Methods

### 3.1. Materials

The gene–trait associations were mainly collected from published papers related to the large yellow croaker. The database contained information about the relevant genes, the corresponding traits that have been generated, and the causes for the trait (e.g., genes of *PPARa* [13], *COX2* [15], *IL-1β* [16] and traits of adipose [17], defense [18], and growth [19], respectively). As a result, 77 gene–trait associations (including 24 large yellow croaker traits and 102 genes) were collected from more than 1000 papers (see Supplementary Materials). We constructed a gene–trait matrix of size of 102 × 24 as the information source, among which 1 represented the gene and trait which have an association.

### 3.2. Methods

#### 3.2.1. Problem to Formalize [8]

**Figure 2:**
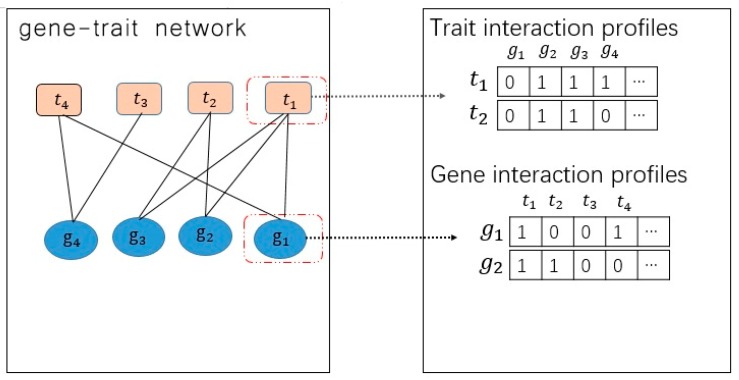
Description the use of a known gene–trait network to obtain trait interaction profiles, gene interaction profiles and the corresponding binary adjacency matrix *A*. The orange node represents the trait, and the blue node represents the gene in the network.

We needed to determine how to predict a new link based on a known gene–trait association network. We assumed that set $X_{g}=\left\{g_{1},g_{2},g_{3}\ldots g_{i}\right\}$ and $X_{t}=\left\{t_{1},t_{2},t_{3}\ldots t_{i}\right\}$ represented genes and traits, respectively. After collecting existing data, some associations between genes and traits were determined, which helped us build a heterogeneous network to express connections between genes and traits. In this network, we treated genes and traits as nodes and connections as edges. We used binary adjacency matrix to express this network: if $A_{ij}$ was 1, then gene *i* and trait *j* were connected, or they were not (see Figure 2).

#### 3.2.2. Gaussian Interaction Profile Kernel Similarity for Genes

We made the following assumption before our experiments [20]: that functionally similar genes share the same pattern. If a gene *g* and a trait *t* are related, then genes functionally similar to gene *g* are more likely to be related, with traits similar to the specific trait *t*. The Gaussian interaction profile kernel similarity for genes was constructed from a known gene–trait association network. We used a similar assumption with relation to genes. By using genes, we proposed that the interaction profile $IP\left(g_{i}\right)$ was the binary vector encoding the existence or lack of interaction with every target in the considered gene–trait network [8]. Given a gene *i*, the interaction profile $IP\left(g_{i}\right)$ would be defined as the *ith* column of the matrix A. The interaction profiles extracted from a gene–trait interaction network can be used as feature vectors for a classifier. Figure 2 illustrates the construction of interaction profiles [2]. Then, based on the distribution of the relationship between the gene and the trait, we used the following formula to calculate the Gaussian interaction profile kernel similarity, *KG*, between each gene pair (i.e., $g_{i}$ and $g_{j}$):

(1) $$KG(g_{i},g_{j})=\exp\left(-\gamma_{g}{\left\|IP(g_{i})-IP(g_{j})\right\|}^{2}\right)$$

(2) $$\gamma_{g}=\gamma_{g}^{\prime }/\left(\frac{1}{n_{g}}\sum_{k=1}^{n_{g}}{\left\|IP(g_{k})\right\|}^{2}\right)$$

where $\gamma_{g}$ is the normalized kernel bandwidth based on the new bandwidth parameter $\gamma_{g}^{\prime }$; $n_{g}$ is the number of genes; and $KG(g_{i},g_{j})$ is the Gaussian interaction profile kernel similarity between gene $g_{i}$ and $g_{j}$.

#### 3.2.3. Gaussian Interaction Profile Kernel Similarity for Trait

**Figure 3:**
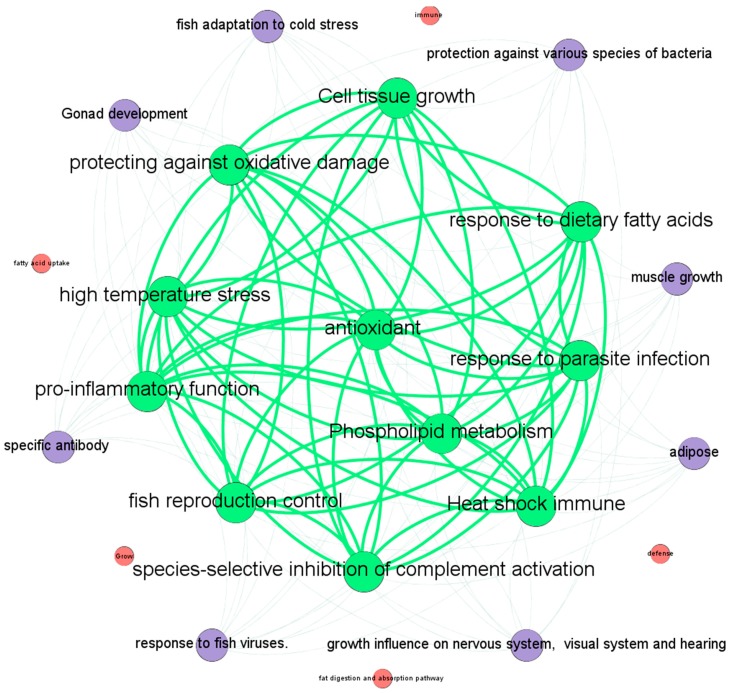
The similarity network between traits, where the nodes represent traits, the weighted edges represent the similarity associations between nodes, the size of a node corresponds to the number of neighbors of the node, and nodes in the network with identical colors have the same similarity value.

By making an analogy between the calculating methods of gene associations and trait associations, we can use the same method to obtain connections of traits. The Gaussian interaction profile kernel similarity for traits (*KT*) was computed in a similar way as for genes, as follows:

(3) $$KT(t_{i},t_{j})=\exp\left(-\gamma_{t}{\left\|IP(t_{i})-IP(t_{j})\right\|}^{2}\right)$$

(4) $$\gamma_{t}=\gamma_{t}^{\prime }/\left(\frac{1}{n_{t}}\sum_{k=1}^{n_{t}}{\left\|IP(t_{k})\right\|}^{2}\right)$$

where $\gamma_{t}$ is the normalized kernel bandwidth based on the new bandwidth parameter $\gamma_{t}^{\prime }$; $n_{t}$ is the number of traits; and $KT(t_{i},t_{j})$ is the Gaussian interaction profile kernel similarity between trait $t_{i}$ and $t_{j}$. A relationship network of all traits of similarity is presented in Figure 3. According to the experimental results, and in order to better display the similarity of the traits in the graph, we kept similarity correlations of greater than 0.35.

#### 3.2.4. KATZ-YC

KATZ measure has been successfully applied in many areas, such as link prediction of some microbial-diseases [20] associations, social network prediction [6], and the prediction of lncRNA-disease [21] associations. KATZ measure is a web-based approach that predicts the relevance of related nodes in a network by calculating the similarity between nodes in a heterogeneous network [20]. In this paper, we proposed a new model based on the KATZ algorithm in order to explore the association of genetic traits in the large yellow croaker (*Pseudosciaena crocea*) [22]. The KATZ method calculates the similarity between nodes based on the number and length of paths. Thus, we calculated the relationships between the genes and traits of the large yellow croaker in such a way as to represent them as the number of paths between genes and traits in heterogeneous networks. Similarities between nodes would be affected by number of steps and step length, which means that shorter distances indicate a high possibility of correlations and longer ones indicate a low possibility of correlations. Therefore, we incorporated steps and step lengths as effective information for the process of predicting possible associations between genes and traits. This method considers both the quantity and length of paths between nodes in a graph as valid similarity metrics. Thus, we transformed the problem of measuring gene–trait associations by counting the number of walks of connections between each gene and each trait in the heterogeneous network. Heterogeneous networks consist of genetic similarity networks and feature similarity networks. The genetic similarity network was based on the gene Gaussian interaction profile, and the trait similarity network was based on the trait Gaussian interaction profile. The known gene–trait association network was constructed based on the collected databases and literature.

The KATZ-YC first calculated the number of walks between gene nodes (i.e., $g_{i}$) and trait nodes (i.e., $t_{i}$) in the known gene–trait association network. As mentioned, we stored the gene–trait association heterogeneous network in an adjacent matrix A. We calculated $A_{\mathrm{ij}}^{l}$ to obtain the number of l-length walks between $g_{i}$ (gene) and $t_{i}$ (trait). By using the Gaussian interaction profile kernel, we obtained homogeneous networks of similarities in genes and traits. In order to implement the similarities of genes and traits into prediction of links related to genes and traits, we further integrated the gene similarity matrix *KG*, the trait similarity matrix *KT*, and the adjacent matrix *A* as follows:

(5) $$A^{*}=\left[\begin{array}{cc} KT & A\\ A^{T} & KG \end{array}\right]$$

The new synthesis matrix A* here can be used to further predict gene–trait associations, which allows the application of the KATZ-YC to new genes and traits with unknown associations if we could acquire further similarity information without relying on known gene–trait network topology information. All walks of different lengths were integrated in order to obtain a single measurement of each gene–trait pair. However, taking into account the different contributions of different steps, greater similarity was obtained between the nodes that are closer, whereas the correlation between nodes with longer steps may be smaller. Thus, we introduced a nonnegative coefficient Sequence $\partial_{l}$ to control the effect of longer steps. The corresponding coefficients of shorter walks in $\partial_{l}$ would be larger than those corresponding to longer walks. We supposed that the step between the pair of nodes was $l_{1}$ and that $l_{2}$ ($l_{1}$ < $l_{2}$) corresponded to $\partial_{l_{1}}$ > $\partial_{l_{2}}$.

Therefore, we proposed the entity $S(g_{i,}t_{i})$ of matrix S to calculate the potential association probability between gene $g_{i}$ and trait $t_{i}$, as follows:

(6) $$S(g_{i},t_{j})=\sum_{l=1}^{k}\partial_{l}A^{*l}(i,j)$$

Here, we replaced $\partial_{l}$ by $\partial^{l}$ and transformed this equation into the matrix form:

(7) $$S=\sum_{l\geq 1}\partial^{l}A^{*l}={\left(I-\partial A^{*}\right)}^{-1}-I$$

Here, matrix *S* with dimensions of (24 + 102) × (24 + 102) depicted the association possibilities of all the gene–trait pairs. Furthermore, we represented the partitioned matrix $A^{*}$ in a form similar to matrix *S*′ as follows:

(8) $$S^{\prime }=\left[\begin{array}{cc} S11 & S12\\ S21 & S22 \end{array}\right]$$

We finally predicted the result for matrix *S* within the matrix *S*12, which could provide the correlative probability between each gene and trait. However, we collected a only small amount of data because the gene–trait association network is still sparse. Walks with long lengths in a sparse network may be unimportant and could perturb the link prediction. Thus, we set parameter *k* to be 2, 3 and 4 and evaluated the parameter k’s influence on the prediction performance. We calculated the respective unknown association probability when *k* was set as 2, 3 and 4 by the following formula. Here, the final prediction result matrix could be represented by matrices *A*, *KT*, and *KG*.

(9) $$S_{K=2}=\partial \cdot A+\partial^{2}\cdot \left(KT\cdot A+A\cdot KG\right)$$

(10) $$S_{K=3}=S_{K=2}+\partial^{3}(A\cdot A^{T}\cdot A+KM^{2}\cdot A+KG\cdot A\cdot KT+A\cdot KT^{2})$$

(11) $$\begin{array}{l} S_{K=4}=S_{K=3}+\partial^{4}\cdot (KG^{3}\cdot A+A\cdot A^{T}\cdot KG\cdot A+\\ KG\cdot A\cdot A^{T}\cdot A+A\cdot KT\cdot A^{T}\cdot A)+\partial^{4}\cdot (A\cdot A^{T}\cdot A\cdot KT+\\ KG^{2}\cdot A\cdot KT+KG\cdot A\cdot KT^{2}+A\cdot KD^{3}) \end{array}$$

## 4. Discussion and Conclusions

Considering research about gene–trait associations of large yellow croakers was scarce, and that databases of this species were lacking, we achieved the following two tasks in this paper. First, we collected genes related to economic traits of large yellow croakers by searching various literatures and collated expressions, protein sequences, and other information associated with these genes. Second, we used a heterogeneous network to describe gene and trait similarity based on gathered known information about gene–trait associations. We further used that information to implement the KATZ-YC algorithm, improved from KATZ, in order to perform the prediction work on unknown gene–trait associations.

With the development of molecular technology, more correlation between genes and traits will be found. We can build a database by collecting more data based on the abovementioned ones, and we can execute predictive models. Through the association between genes and traits, we have obtained a possible existing link between some economic traits. This work contributes to filling the void in associations between genes and economic traits of large yellow croakers. In the aquaculture of the large yellow croaker, the probability of occurrence of other traits can be predicted according to the appearance of some traits. These traits could effectively promote growth and reproduction or the prevention of possible illnesses. This understanding can further accelerate the development of the large yellow croaker breeding industry.

## Figures and Tables

**Table:** matrix *A*:

	T	*t*_1_	*t*_2_	*t*_3_	*t*_4_	…
G						
*g*_1_		1	0	0	1	…
*g*_2_		1	1	0	0	…
*g*_3_		1	1	0	0	…
*g*_4_		0	0	1	1	…
…		…	…	…	…	…

**Table 1:** Performance comparison among different parameter settings (k = 2, 3, 4) in the framework of LOOCV.

K = 2	K = 3	K = 4
0.7564	0.7766	0.7760

**Table 2:** Performance comparison among different parameter settings (k = 2, 3, 4) in the framework of 2-fold cross validation.

K = 2	K = 3	K = 4
0.7036 ± 0.03	0.7183 ± 0.03	0.7132 ± 0.05

**Table 3:** Performance comparison among different parameter settings (k = 2, 3, 4) in the framework of 5-fold cross validation.

K = 2	K = 3	K = 4
0.7338 ± 0.03	0.7476 ± 0.03	0.7357 ± 0.04
